# Our journey toward implementation of digital breast tomosynthesis in breast cancer screening: the Malmö Breast Tomosynthesis Screening Project

**DOI:** 10.1117/1.JMI.12.S1.S13006

**Published:** 2024-10-24

**Authors:** Anders Tingberg, Victor Dahlblom, Magnus Dustler, Daniel Förnvik, Kristin Johnson, Pontus Timberg, Sophia Zackrisson

**Affiliations:** a Lund University, Medical Radiation Physics, Department of Translational Medicine, Faculty of Medicine, Lund, Sweden; b Skane University Hospital, Radiation Physics, Department of Hematology, Oncology and Radiation Physics, Malmö, Sweden; c Lund University, Diagnostic Radiology, Department of Translational Medicine, Faculty of Medicine, Lund, Sweden; d Skane University Hospital, Department of Imaging and Physiology, Malmö, Sweden

**Keywords:** digital breast tomosynthesis, mammography, breast cancer screening

## Abstract

**Purpose:**

The purpose is to describe the Malmö Breast Tomosynthesis Screening Project from the beginning to where we are now, and thoughts for the future.

**Approach:**

In two acts, we describe the efforts made by our research group to improve breast cancer screening by introducing digital breast tomosynthesis (DBT), all the way from initial studies to a large prospective population-based screening trial and beyond.

**Results:**

Our studies have shown that DBT has significant advantages over digital mammography (DM), the current gold standard method for breast cancer screening in Europe, in many aspects except a major one—the increased radiologist workload introduced with DBT compared with DM. It is foreseen that AI could be a viable solution to overcome this problem.

**Conclusions:**

We have proved that one-view DBT is a highly efficient screening approach with respect to diagnostic performance.

## 1 Prologue

Mammography is a particularly difficult radiological field and requires highly specialized radiologists to achieve acceptable levels of detection and classification of breast cancer without excessive recalls. A prerequisite is therefore images of the highest possible diagnostic quality and acquired at the lowest possible radiation dose because the breast is an organ that is relatively sensitive to radiation,^1^ and most of the women in the screening population are healthy. Digital mammography (DM) is still the gold standard breast cancer screening method in most of Europe, with a sensitivity claimed to be around 80%.^2^ When we in our research group first learned about digital breast tomosynthesis (DBT) during a presentation at the SPIE Medical Imaging symposium in 2004,^3^ it was obvious that this new technique would become a game changer in breast cancer screening.^4^ Even non-radiologists were able to detect breast cancers in selected cases, cancers that were missed in the corresponding DM image.

## 2 Act I: Initial Studies

**Fig. 1:**
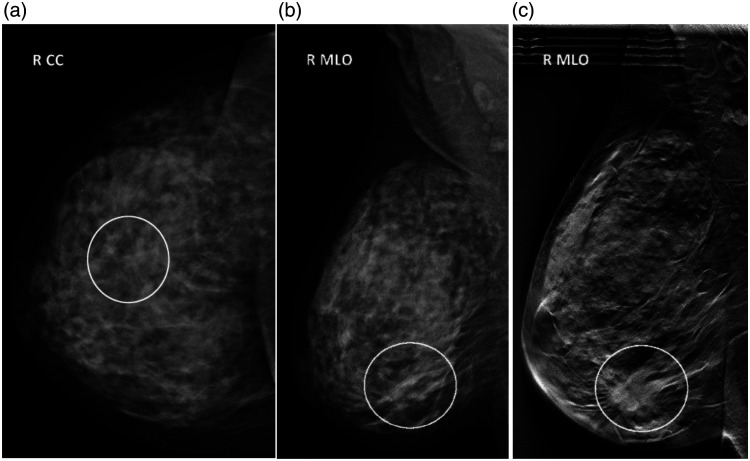
(a) and (b) DM images in the CC and MLO projections, respectively, of the right breast of a 57-year-old woman. (c) The same breast imaged with DBT. Note that the tumor, a 2.8-cm grade 3 invasive ductal carcinoma (marked with a circle), is clearly visible in the DBT image, but difficult to detect in the two mammography images. Reproduced with permission from Ref. 5.

After constructive discussions with representatives from Siemens Healthcare (Erlangen, Germany), it was decided that one of their first DBT prototypes (modified Siemens Mammomat Novation) should be installed in our lab at Skane University Hospital, Malmö, Sweden, in 2006. A number of studies that investigated the potential of tomosynthesis for breast cancer diagnosis compared with mammography were initiated at our lab. Images were collected from women who we believed had the greatest difference in tumor visibility between DBT and DM. In these studies, the inclusion criteria were negative or unclear mammography, in combination with palpable and/or visible lesions on ultrasound (US). The DBT imaging was performed in the view (craniocaudal, CC or mediolateral oblique, MLO) in which the tumor was least visible on DM. If the tumor was only visible on US, the DBT was performed in the MLO view. All available images [including DM, DBT, US, and magnetic resonance imaging (if available)] were collected for these patients together with pathological anatomy diagnosis. The cancers were verified by needle biopsy and pathology and the normal or benign cases with 2-year follow-up. During roughly two and a half years, over 250 cancer cases were collected, forming one of the, at that time, largest collection of tumors imaged with DBT and over 150 normal or benign cases. One of our early examples showing the superiority of DBT compared with DM is presented in Fig. 1. We have used this example in several presentations, and it has convinced even the most skeptical radiologists of the potential of DBT.

From our DBT image database, we selected images of 40 confirmed cancers which we used in a pilot study comparing the image quality, defined as cancer visibility, of one-view DBT to one-view DM, to two-view DM, and to two-view DM + US. The image quality was significantly higher for DBT compared with one-view and two-view DM, whereas there was no difference between DBT and two-view DM + US in this study.^5^ Please note that the DBT images were one-view and in most cases (92.5%) in the MLO projection.^5^

Compression of the breast during mammographic imaging serves multiple purposes. First, it immobilizes the breast which reduces motion artifacts. Second, the spread of the breast tissue reduces the overlapping of the tissue which may hide tumors. Third, the reduced breast thickness limits the radiation dose to the breast.^6^^,^^7^ However, some women refrain from their screening examination due to the pain caused by the compression.^8^

As DBT is an imaging technique with depth resolution, it may benefit from reduced compression (and a slightly increased compressed breast thickness). To explore this effect, a study was carried out in which we investigated the image quality at normal compression force compared with half of the normal compression force. Forty-five women were imaged at the two compression levels and with the same acquisition parameters. The results of the study showed no significant difference in image quality between the two compression levels.^9^ The majority of the women felt that half compression was more comfortable than the standard compression.^9^

In parallel, we investigated whether breast cancer size and stage could be more accurately assessed with DBT than with DM and US preoperatively. Radiologists measured the tumor extent of 73 breast cancers on DBT, DM, and US and compared the results to pathological size in the resected breast specimen. The tumor outline could be determined in significantly more cases with DBT (86%) compared with DM (67%) and correlated better with pathology (mean deviation from pathology was 1.5 mm for DBT versus 2.2 mm for DM). The study suggested that DBT is superior to DM in assessing preoperative tumor size and hence the T stage of breast cancer and particularly so in women with dense breasts.^10^

## 3 Act II: Screening with Digital Breast Tomosynthesis

Sweden has a national screening program that invites all women aged 40 to 74 years to screening with DM every 18 or 24 months, depending on age.^11^ The participation rate is around 80%. In Sweden and in Europe, mammography is double-read, meaning that two radiologists independently evaluate the images, and a decision on whether to recall the woman for further investigation is reached. If the opinion of the radiologists is discordant, either a consensus discussion or preferably an arbitration should be carried out.^6^

Encouraged by the positive results of DBT in our early studies, we started planning a prospective population-based screening trial comparing DBT with DM, the Malmö Breast Tomosynthesis Screening Trial (MBTST). The prototype DBT machine was replaced by a commercial machine (Mammomat Inspiration, Siemens Healthcare GmbH, Erlangen, Germany), which could operate in both DM and DBT modes (wide-angle). A random selection of the women invited to attend national breast cancer screening in Malmö also got an invitation to participate in the trial. The women were imaged with two-view DM (CC and MLO projections) and one-view DBT (MLO projection) on a single occasion. The DBT images were acquired with the same beam quality as the DM images, and the automatic exposure control was set to give an average glandular dose of 1.2 mGy per image for DM and 1.6 mGy for one-view DBT (for a standard breast simulated by a 45 mm polymethyl methacrylate phantom^6^). The radiation dose for one-view DBT was thus around 70% of the dose for two-view DM. The DBT images were acquired with 40% less compression force compared with DM (for the MLO projection), motivated by our earlier image quality assessment of reduced compression. The images from the two modalities were evaluated in two different reading arms, meaning that our trial was the first ever that truly investigated the performance of DBT as a stand-alone modality compared with DM, whereas all other studies up to that time had investigated the added value of DBT, i.e., DBT + DM (or synthetic mammography) versus DM.

Our trial included 14,848 women and lasted between January 2010 and February 2015. The results of the trial showed a 34% higher cancer detection rate with DBT compared with DM (8.7 cancers versus 6.5 cancers per 1000 screened women, p<0.0001), at a slightly higher recall rate (3.6% versus 2.5%) which is well below the acceptable highest level recommended in the European guidelines.^6^^,^^12^^,^^13^ The increased cancer detection rate was shown in all breast density categories and not only the densest category, even though the added value of DBT was highest in women in the densest categories.^14^ The mean size of the cancers detected with DBT was smaller than the mean size of the ones detected by DM, indicating earlier detection. The biological profile of the additional cancers detected with DBT was similar to those detected with DM, which included cancers with the least favorable prognosis.^15^ The higher recall rate can partly be explained by the lack of reader experience. We observed that the number of false positives associated with DBT decreased with increasing reader experience and could be expected to decrease further in a situation in which screening with DBT is repeated and with previous DBT examinations available for comparison at screen reading.^13^

An investigation of the interval cancer rate of the women who participated in the MBTST was performed and compared with an age- and screen date–matched control groups that were screened with DM in parallel, at the same location. The analysis showed that the interval cancer rate for the MBTST women was significantly lower, 1.6 per 1000 screened women, compared with 2.8 per 1000 screened women in the control group^16^ giving 40% lower odds for the women getting an interval cancer following DBT screening. A recent follow-up study of the MBTST cohort revealed lower than normal overall detection rates of less aggressive breast cancer subtypes as well as invasive cancer detection at the following consecutive regular DM screening round, further strengthening the benefit of tomosynthesis screening due to earlier detection of clinically relevant invasive cancers.^17^

Several papers with a focus on the clinical experiences and lessons learned from the trial have been published and investigated, e.g., reasons for and types of false-positive lesions,^18^^–^^21^ reasons for non-detection at DBT screening,^22^ and optimization of reading strategies of DBT.^23^

### 3.1 Conclusions from the Screening Trial

One-view DBT has a significantly higher cancer detection rate than two-view DM. The compression force with DBT can be reduced compared with DM. The radiation dose of one-view DBT is comparable to two-view DM. To the best of the authors’ knowledge, all other screening trials comparing DBT to DM have been performed with two-view DBT and in combination with either DM or synthetic mammograms. Even if other studies had more extensive imaging protocols (i.e., two-view DBT), the MBTST results were at the same or better performance level.^24^ We have hence proved that one-view DBT is a highly efficient screening approach with respect to diagnostic performance.

## 4 Epilogue

Although the diagnostic performance of DBT is significantly better compared with DM and conditionally recommended over DM by the European Commission Guidelines,^25^ DM is still the recommended screening modality in Sweden and many other European countries. The disadvantage of DBT is the longer reading time (around 70% longer^26^^,^^27^) which is an obstacle to implementing DBT in resource-starved breast cancer screening programs, especially as the availability of radiologists is already a problem in many countries. Efforts have been made to include AI in the screening workflow to reduce the workload of radiologists. We have studied different scenarios, for example, letting AI replace one reader in a double-reading scenario or excluding low-risk cases from reading.^28^ Further, we have studied if AI can selectively add DBT in high-risk cases in a primarily DM-based screening situation.^29^ These studies showed that with AI, it is possible to maintain or even improve the sensitivity of breast cancer screening with DBT without increasing the radiologist workload.

The trial examinations now constitute parts of the Malmö Breast Imaging (M-BIG) database for further collaborative work on, e.g., AI and DBT.^30^ Introducing DBT in national breast cancer screening programs in Europe seems to benefit from being performed in conjunction with a simultaneous introduction of AI to take advantage of the higher diagnostic performance of DBT without increasing the workload of radiologists.

## Data Availability

Data sharing is not applicable to this article, as no new data were created or analyzed.
